# Is there a pension penalty for late-career family caregiving in Germany? The role of pension care credits and employment career

**DOI:** 10.1007/s10433-026-00923-y

**Published:** 2026-07-14

**Authors:** Ulrike Ehrlich, Nadiya Kelle, Alberto Lozano Alcántara, Laura Romeu Gordo

**Affiliations:** German Centre of Gerontology (DZA), Berlin, Germany

**Keywords:** Family care, Late career, Pension entitlements, Employment trajectories, Life course

## Abstract

Unpaid care for ill, disabled, or frail older family members (hereafter ‘family care’) is most common in late career. Because caregiving can be difficult to reconcile with paid work, it may reduce pension contribution records and later-life financial well-being. Many European countries therefore provide pension care credits, yet eligibility is selective and many caregivers remain uncredited. Against this background, we examine how late-career pension accumulation differs between non-caregivers, credited caregivers, and uncredited caregivers across different late-career employment trajectories. Detailed information on pensions, family care and employment of individuals between the ages of 55 and 65 (N = 814), covering those born between 1946 and 1955, is derived from SOEP-RV, a novel data linkage of administrative records of the German pension insurance (RV) with the German Socio-Economic Panel (SOEP, waves 2001–2020). By means of linear regressions, preceded by sequence and cluster analyses to typify late-career employment trajectories, we found no statistically significant associations between uncredited low-intensity care and lower pension accumulation. Uncredited high-intensity caregivers accumulate fewer pension entitlements within full-time and part-time trajectories. Moreover, credited caregivers accumulate more pension entitlements than non-caregivers, but only within part-time, homemaking, and early retirement trajectories. These findings suggest that the German pension care credit scheme provides selective and uneven compensation and that uncredited high-intensity caregivers tend to face pension penalties.

**Supplementary Information:**

The online version contains supplementary material available at 10.1007/s10433-026-00923-y.

## Introduction

Due to population ageing, the number of people in need of care is expected to increase. In most European countries, care for ill, disabled, or frail older people is often provided unpaid within families or among friends (hereafter ‘family care’) and predominantly by women (e.g. Brandt et al. 2009; Luppi and Nazio 2019). Although family care needs can occur at any point in an individual’s life course (Moen et al. 1994), their prevalence is highest during the caregivers’ *late-career* life stage, i.e. their fifties and sixties, when many parents(-in-law) develop care needs (e.g. Patterson and Margolis 2019). In European countries, on average 13% of people aged 50 and over assist family members or friends dealing with a long-term health problem, old-age frailty, disability, or functional limitation at least on a weekly basis (OECD 2019).

Because time resources are limited, late-career family care can be difficult to reconcile with paid work, potentially leading to fragmented employment careers. Yet in most Western welfare states, adequate pensions are largely determined by continuous full-time employment (earnings), whereas part-time work or periods of non-employment are associated with lower later-life financial well-being (e.g. Fasang et al. 2013; Madero-Cabib and Fasang 2016; Sefton et al. 2011). Family caregiving may therefore generate fragmented pension contribution records through its impact on employment careers. To acknowledge these risks, several European countries have introduced care credit schemes that aim to compensate for reduced pension contributions, signalling growing social policy attention to family care-related pension penalties (Van den Bosch et al. 2023; Eggers and Grages 2023).

While many studies examine the short-term labour market consequences of late-career family caregiving, such as changes in working hours, labour market participation, or wages (e.g. Glauber 2019; Lilly et al. 2007; Moussa 2019; Van Houtven et al. 2013), the longer-term implications for pension outcomes are rarely addressed. However, such short-term associations do not allow direct inferences about pension outcomes, which reflect cumulative contributions over time and are shaped, among other factors, by selective access to care credits and longer-term employment careers. The few existing studies examining longer-term financial consequences have either relied on broad indicators of later-life economic well-being (e.g. poverty risk) (Wakabayashi and Donato 2006) or approximated pension outcomes using retrospective work and caregiving history data (Evandrou and Glaser 2003), rather than measuring pension entitlements directly. More recent research using administrative pension contribution data provides more precise evidence of care-related pension effects (Czaplicki 2016; Rothgang and Unger 2013). However, this research focuses exclusively on caregivers who receive care credits under a compensatory care credit scheme. Because care credits are granted only under specific eligibility conditions (Van den Bosch et al. 2023; Eggers and Grages 2023), this approach sheds limited light on pension consequences among the large share of family caregivers who remain outside these schemes, namely those providing ‘uncredited’ family care. Taken together, this leaves a critical empirical gap in understanding how family caregiving shapes pension accumulation through both credited and uncredited pathways. Addressing this gap requires a life-course perspective that considers paid work and family life to be *interdependent* and *path-dependent* (Bernardi et al. 2019; Elder 2009; Mayer 2015). In late career, caregiving involvement, work-care reconciliation strategies and post-care labour market behaviour are likely to differ across employment trajectories, with implications for pension accumulation (e.g. Bertogg and Settels 2023; Carmichael and Ercolani 2016; Czaplicki 2020).

Accordingly, we ask: (1) How does late-career pension contribution accumulation differ between non-caregivers, credited caregivers, and uncredited caregivers across different late-career employment trajectories? (2) To what extent do pension care credits compensate for caregiving-related differences in pension contribution accumulation across late-career employment trajectories?

To address these research questions, we draw on SOEP-RV data (Lüthen et al. 2022), a novel linkage of survey data from the German Socio-Economic Panel (SOEP; Goebel et al. 2019) with administrative records from the German public pension insurance (*Rentenversicherung;* RV). This linkage allows us to distinguish between credited and uncredited caregiving and to examine how each relates to pension contribution accumulation across nuanced late-career employment trajectories.

## Background

### Pensions, paid work and family care in Germany

Germany, the national context of this study, is a conservative welfare state characterised, among other things, by a Bismarckian employment-centred pension system in which benefits are earnings-related and designed to *conserve* individuals’ pre-retirement standard of living (e.g. Esping-Andersen 1999; Ebbinghaus 2021). The public pension system operates on a pay-as-you-go basis and constitutes the main source of income in later life for most pensioners (BMAS 2024).^1^ Pension entitlements are accumulated through mandatory contributions, which since 2018 amount to 18,6% of gross earnings and are shared equally between employers and employees. These contributions are converted into pension earning points (PEPs), reflecting each insured person’s earnings relative to the national average. One PEP is awarded for earnings at exactly the national average in a given year, while lower or higher annual earnings result in proportionally fewer or more PEPs, up to a yearly cap set by the contribution assessment ceiling (*Beitragsbemessungsgrenze*). In 2025, this ceiling was €96,600, corresponding to a maximum of 1.91 PEPs (Deutsche Rentenversicherung 2026). The total number of accumulated PEPs ultimately determines the final pension level, meaning that longer and more continuous full-time employment leads to higher pensions, whereas career interruptions, part-time work, or low-wage jobs reduce pension entitlements. The total number of accumulated PEPs is then multiplied by the pension value, which indicates the monetary worth. As of July 1, 2025, the pension value was €40.79 (Deutsche Rentenversicherung 2025a). Thus, when family care responsibilities disrupt employment continuity or reduce working hours and earnings, late-career caregiving can lead to a lower accumulation of PEPs, which in turn negatively affects pension entitlements.

At the same time, the extent to which late-career family caregiving impacts on pensions depends on how the German welfare state compensates family care. Germany is recognised for its explicit familialistic care regime, strategically transferring the responsibility of caregiving to the family (Leitner 2003). This is reflected in the fact that in 2023, of the 5.7 million people recognised as needing long-term care according to the long-term care insurance, 86% (4.9 million) were cared for in their home environment, predominantly by family members (Destatis 2024). However, the number of family caregivers cannot be directly inferred from this figure, as care dependents may have multiple caregivers, and many people beyond those meeting the criteria for long-term care insurance benefits also require and receive help, support, or care. In 2023, roughly one quarter of individuals in middle and late working-age reported regularly providing support or care to one or more persons due to health problems, corresponding to approximately 5.5 million people in Germany (Ehrlich et al. 2024), with up to one quarter caring for persons not receiving long-term care insurance benefits (Brandt et al. 2025; Ehrlich and Kelle 2019). Women are disproportionately represented among caregivers and, on average, provide care more intensively and over longer periods than men (Czaplicki et al. 2018; Güneyli et al. 2025).

For individuals caring for a home-based long-term care insurance beneficiary, the German long-term care insurance can, under certain conditions, pay pension contributions into the public pension scheme through a pension care credit system. However, this is only possible if the caregiver spends at least 10 h a week caring for the person in need,^2^ and if the caregiver is not in paid employment for more than 30 h a week. The level of pension care credits depends on the care recipient’s classified care needs (*Pflegegrade*) and the type of care benefits selected (cash benefits and benefits in kind). For individuals providing continuous credited family care for one year in 2023, the level of pension care credits could range between 0.18 and 0.94 PEPs (Deutsche Rentenversicherung 2023, 2024)^3^: a caregiver is treated similarly to an average worker in pension terms only if they provide full-time home-based care for a person with the highest care level (*Pflegegrad*) continuously over an entire year without additional professional care services—an extremely rare scenario, as this level and care situation represent only about 1.4% of all care dependents (Destatis 2024). In 2023, a total of 1.1 million caregivers received pension care credits, 86% of whom were women (Deutsche Rentenversicherung, 2025b). Although these figures are not specific to a particular life stage, they clearly fall well below the estimated number of caregivers in mid-to-late working age, suggesting that many caregivers remain uncredited—either because they are unaware of the scheme, do not apply, or do not qualify for it. Germany therefore offers a well-suited case for examining late-career pension accumulation among non-caregivers, credited caregivers, and uncredited caregivers. The extent to which care credits offset, reduce, or potentially exacerbate pension disadvantages is complex and depends, among other factors, on how individuals combine family care and paid work.

### Previous research

#### Family care and paid work in late careers

Life-course research highlights that employment trajectories are strongly *interdependent* with family responsibilities, as both draw on individuals’ limited time resources (Bernardi et al. 2019; Mayer 2015). This *interdependence* implies a two-way relationship. On the one hand, late-career family caregiving may influence paid work trajectories and thus pension accumulation. On the other hand, paid work may influence whether individuals take up family care and how intensively they provide it, introducing *selection* processes with implications for pension accumulation.

Empirical research on the short-term labour market consequences of family caregiving yields mixed findings. While some studies report modest reductions in working hours associated with caregiving responsibilities (e.g. Leigh 2010; Van Houtven et al. 2013), others find no such effects (e.g. Keck 2012). Findings at the extensive margin are similarly inconsistent: some studies report an increased likelihood of labour market withdrawal among caregivers (e.g. Heitmueller 2007; Leigh 2010), whereas others do not (e.g. Meng 2013; Van Houtven et al. 2013). A more consistent pattern emerges, however, when care intensity, typically measured in hours, is taken into account. These studies indicate that adverse employment effects arise primarily among individuals who devote substantial time to family care (e.g. Berecki-Gisolf et al. 2008; Ehrlich 2023; Lilly et al. 2007; Kelle 2020; King and Pickard 2013; Moussa 2019). Overall, these findings suggest that negative labour market outcomes are not universal, although most studies point to adverse outcomes among high-intensity family caregivers.

Beyond short-term labour market responses, the life-course principle of *interdependence* highlights that paid work may also shape whether and how intensively individuals provide care. This is particularly important in the late-career life stage, when *path dependencies* have already channelled individuals’ family and work lives into specific trajectories (e.g. Schmitz et al. 2023). While studies based on short-window longitudinal data find no evidence that time spent in paid work just prior to caregiving predicts caregiving uptake (Berecki-Gisolf et al. 2008; Pavalko and Artis 1997), studies based on sequenced long-term longitudinal data suggest that careers characterised by labour market detachment are associated with more intensive and longer caregiving (Carmichael and Ercolani 2016; Czaplicki 2020). Thus, caregiving uptake may be largely independent of current employment status, whereas the intensity of care provided depends on the path-dependent employment career. Moreover, prior employment careers are likely to shape work–care reconciliation strategies, with individuals in full-time careers making different adjustments than those in part-time or homemaking trajectories (e.g. Bertogg and Settels 2023; Czaplicki 2020). They may also condition post-care labour market outcomes; for example, individuals with more career interruptions are less likely to return to work after caregiving ends (Keck 2016). This underscores the value of a within-employment-trajectory perspective for understanding how late-career caregiving relates to cumulative pension accumulation: care intensity, work–care reconciliation strategies, and post-care employment behaviour are likely to vary across employment careers, with implications for pension accumulation.

#### Family care and its pension implications

Evidence on the link between late-career family caregiving and pension contributions is scarce. Existing studies in the German context draw primarily on administrative pension contribution data and are therefore restricted to caregivers who apply for, qualify for, and receive pension care credits. While these studies identify compensatory mechanisms and even positive effects of caregiving on pension entitlements (Czaplicki 2016; Rothgang and Unger 2013; Söhn and Mika 2017), they cannot draw conclusions about caregivers who do not receive such credits. Moreover, within the credited group, evidence suggests heterogeneity by employment attachment during caregiving. Czaplicki (2016) distinguishes caregivers who combine the caregiving period with employment subject to social security contributions for more than 50% of the period, for less than 50%, or not at all. Findings suggest that pension losses are unlikely among credited caregivers overall. Moreover, the reported effects vary across these groups: compensation appears stronger among caregivers who spend a smaller share of the caregiving period in socially insured employment, while pension entitlements may even increase among those who combine caregiving with socially insured employment for most of the period. However, this evidence is confined to credited caregivers and based on relatively coarse groupings defined by the share of the caregiving period spent in employment subject to social security contributions.

### The present study

This study examines how late-career family caregiving is associated with pension contribution accumulation by comparing non-caregivers, credited caregivers, and uncredited caregivers across late-career employment trajectories. It uses direct measures of pension entitlements and captures caregiving situations that extend beyond those covered by pension care credits. The analysis addresses heterogeneity in both caregiving and employment by differentiating care intensity and credited vs. uncredited caregiving within nuanced late-career employment trajectories that may condition care involvement, work-care reconciliation strategies, and post-care labour market attachment, with implications for cumulative pension accumulation. Finally, it situates these associations within the institutional context of the German pension and long-term care systems, which shape eligibility for care credits and how work-care interdependencies translate into pension accumulation.

## Data and methods

### Data

We use panel data from the SOEP-RV (Forschungsdatenzentrum der Rentenversicherung, 2024; Lüthen et al. 2022). SOEP-RV links survey information from the German Socio-Economic Panel (SOEP; Goebel et al. 2019) with administrative information from the German pension insurance (RV). The linked data offer significant advantages for studying the relationship between family care and PEP accumulation for individuals within different late employment careers. First, we have detailed information on individuals’ accumulated PEP contributions (RV data). Second, we have information on credited family care, referring to care that generates pension entitlements (RV data). Third, we have information on individuals’ self-reported family caregiving (SOEP data), which we can classify as credited or uncredited when combined with pension care credit information from the RV records. Fourth, by exploiting both SOEP and RV data, we have accurate information on individuals’ late-career employment trajectories, allowing us to differentiate between episodes of full-time, part-time, and marginal employment, unemployment, homemaking, early retirement due to occupational disability or reduced earnings capacity, and old-age retirement. Extensive data management and harmonisation processes were required to set up a ready-to-analyse dataset.

*Sample.* For the purpose of this study, we use SOEP-RV data from 2001 to 2020, as family care started to be surveyed in SOEP in 2001. Within this period, we restrict our sample to those aged between 55 and 65 (n = 6,096) for whom we have detailed monthly family care and employment information for the majority of the observation window.^4^ This results in a sample of individuals born between 1946 and 1955, for whom the statutory retirement age is 65, which defines the upper age limit of 65 as the end of the late-career phase. This selection excluded observations of n = 5,124 individuals. We further excluded observations of n = 127 individuals who were identified as civil servants or self-employed for most of their careers because they are not covered by public pension insurance, or only to a low degree, and may bias our analyses (see Figure S1.1 in Section S1 of the Supplementary Material detailing all sample selection steps). After these selections, our analytical sample comprises n = 814 individuals, amounting to 98,494 person-month observations (121 person-month observations per individual). This 10-year-long person-month information allows us to aggregate monthly observations between ages 55 and 65 into person-level measures of pension contributions, family care, and late-career employment trajectories up to age 65, as shown below.

*Sample limitations.* Two limitations should be noted. First, using the linked SOEP-RV data comes with the limitation that not all SOEP respondents were asked to consent to record linkage with RV data; and not all of those asked consented (see Lüthen et al. 2022). This implies that the SOEP-RV sample may not be representative for the entire German population. Furthermore, by using a sample of individuals with nearly complete employment histories and care information between the ages of 55 and 65, our analytical sample may be biased due to higher panel attrition in certain groups, particularly as individuals providing high-intensity family care are more likely to drop out of panel surveys (e.g. Rothenbühler and Voorpostel 2016), which may lead to an underrepresentation of high-intensity caregivers. To assess potential selectivity by consent to record linkage and subsequent sample restrictions, Table S2.1 in the Supplemental Material compares key characteristics at age 55 between the analytical sample, the unrestricted SOEP-RV sample and the full SOEP-Core sample. Overall, the differences are moderate. The analytical sample is characterised by a slightly higher share of female respondents and a stronger concentration in West Germany. Moreover, it is somewhat more highly educated and has accumulated more PEPs at age 55. Thus, our analytical sample is slightly positively selected with respect to labour market attachment and PEP accumulation. Consequently, our results should be interpreted cautiously, as individuals with higher education and stronger labour market attachment may have greater scope to combine employment and family care, potentially leading to different caregiving-related pension outcomes than in the broader population. Second, due to the limited number of cases, gender differences can be accounted for only to a limited extent. This is relevant given well-documented gendered patterns in family caregiving and labour market participation.

*Dependent variable.* The dependent variable is the aggregated sum of the monthly PEPs accumulated between ages 55 and 65, capturing individuals’ total late-career pension contribution accumulation at age 65 at the person level.

*Independent variables.* The first set of independent variables captures individuals’ family care provision between ages 55 and 65, encompassing both credited and uncredited caregiving. We differentiate three continuous family care measures: (1) ‘credited care’, (2) ‘uncredited low-intensity care’ (≤ 10 h per week), and (3) ‘uncredited high-intensity care’ (> 10 h per week). All three measures count the total number of months between ages 55 and 65 in which a respondent provided the respective type of care. Information on measure (1) is drawn from the RV data and is based on individuals’ person-month pension contribution records. Credited care can be identified because the long-term care insurance of the care recipient credits the caregiver’s pension account. Information on measures (2) and (3) comes from the SOEP and relies on respondents’ self-reports of the number of hours spent on a typical workday providing care and support for individuals in need of care. As care is reported annually in the SOEP but needs to be aligned with person-month data, caregiving spells are assigned from the fifth month before to the sixth month after the interview month and extended across waves if reported consecutively. We assess the sensitivity of this approach using shorter caregiving periods, as described in the Robustness Checks section. Family care measures (2) and (3) are classified as ‘uncredited’ if no corresponding pension care credits are recorded in the RV data for the same month; when both sources indicate caregiving in a given month, credited care takes precedence. As a result, in each month an individual can engage in only one type of family care or none at all. Accordingly, by age 65, the combined total of the three family care measures can range from 0 to 121 months. Within ‘uncredited care’, we distinguish between (2) low- and (3) high-intensity care using a threshold of 10 h per week. Weekly care hours are calculated by multiplying typical workday care hours by five. This threshold follows prior research showing that more than 10 weekly family care hours are associated with substantial labour market adjustments (e.g. Ehrlich 2023; Lilly et al. 2007; Kelle 2020; Moussa 2019). In the German context, it is particularly relevant, as the long-term care insurance uses ≥ 10 weekly care hours, alongside additional criteria, as a requirement for granting pension care credits (see Sect. "Pensions, paid work and family care in Germany"). As some individuals meet the weekly hours criterion but not the remaining requirements, (3) uncredited high-intensity care captures time-intensive caregiving without entitlement to pension care credits.

A further key independent variable in our study mirrors sample members’ employment careers between ages 55 and 65. Based on a cluster sequence analysis of information on monthly employment statuses (see Section S3 in the Supplementary Material for a detailed description of the method and the employment variables), we identified five late-career employment clusters (see Table 1). In line with the life-course perspective, this approach accounts for the timing, ordering, and stability of employment states rather than reducing late careers to single indicators such as dominant employment status or hours worked. The resulting clusters therefore represent employment trajectories within the same late-career life stage (ages 55–65), allowing caregivers and non-caregivers to be compared within structurally similar work-life contexts.

**Table 1:** Description of the clusters

	Late-career employment clusters				
	Full-time employment	Part-time employment	Unemployment/Marginal employment	Early retirement	Homemaker
*Share of time spent btw. 55–65 years of age in each status (in %)*					
Old-age retirement	12.5	21.5	8.3	58.4	3.0
Occupational disability/ reduced earnings capacity	0.1	1.0	0.2	12.5	0.0
Full-time employment	80.5	9.7	1.6	16.0	0.0
Part-time employment	0.9	59.0	5.8	1.5	2.3
Marginal employment	0.6	2.2	43.0	2.0	4.6
Unemployment	3.5	4.1	36.6	6.3	0.5
Homemaking	0.9	1.6	4.0	2.3	89.2
Other	0.9	0.9	0.5	1.0	0.3
*N (Share)*	*284 (34.9%)*	*161 (19.8%)*	*86 (10.6%)*	*222 (27.3%)*	*61 (7.5%)*

Source: SOEP-RV 2020 v.2.0, own computations

Cluster 1 (‘full-time employment’, 34.9%) is primarily characterised by full-time employment followed by old-age retirement. Cluster 2 (‘part-time employment’, 19.8%) is primarily comprised of individuals employed part-time before entering old-age retirement. Cluster 3 (‘unemployment/marginal employment’, 10.6%) is dominated by long episodes of unemployment and marginal employment. Cluster 4 (‘early retirement’, 27.3%) is mainly composed of individuals retiring before age 65, often from full-time employment or due to occupational disability or reduced earnings capacity. Cluster 5 (‘homemaker’, 7.5%) mainly comprises individuals engaged in homemaking with some episodes of marginal employment. These five clusters are treated as a categorical variable in our analyses.

*Control variables.* To ensure that alternative explanations for the level and speed of PEP accumulation are taken into account, we also consider the following control variables: gender, region where the individual has lived longest between the ages of 55 and 65 (West or East Germany), education level, phased retirement,^5^ retirement timing, and number of months with missing information on family care. At the same time, gender and education may affect not only PEP accumulation, but also the likelihood of being in a particular employment cluster or family care take-up. Table 3 in the Appendix presents a description of the operationalisation of all variables.

### Method

We estimate a multivariate ordinary least squares (OLS) regression model with robust standard errors. Both the dependent variable and the independent variables are cumulative measures; accordingly, the analysis is conducted on individual-level aggregates. We explore the relationship between the three family care measures (months spent in uncredited low-intensity care, uncredited high-intensity care and credited care) and the total number of PEPs accumulated between ages 55 and 65. Our model includes interaction terms of the three family care measures and the employment clusters. This allows us to examine how the relationship between family care and PEP accumulation varies across individuals within different late-career employment clusters. The model was adjusted for the control variables detailed above.

The model specification is:

$${PEP}_{i}=\alpha +{\beta}_{1}{ULC}_{i}+{\beta}_{2}{UHC}_{i}+{\beta}_{3}{CC}_{i}+{\sum}_{k=2}^{5}{\gamma}_{k}{Cluster}_{ik}+ {\sum}_{k=2}^{5}{\delta}_{k}\left({ULC}_{i} \times {Cluster}_{ik}\right)+ {\sum}_{k=2}^{5}{\phi}_{k}\left({UHC}_{i} \times {Cluster}_{ik}\right)+ {\sum}_{k=2}^{5}{\psi}_{k}({CC}_{i} \times {Cluster}_{ik}) +\theta {X}_{i}+ {\varepsilon}_{i}$$

where *PEP*_*i*_: total accumulated pension entitlements between ages 55 and 65, *ULC*_*i*_: cumulative months of uncredited low-intensity family care, *UHC*_*i*_: cumulative months of uncredited high-intensity family care, *CC*_*i*_: cumulative months of credited family care, *Cluster*_*ik*_: dummy variable indicating employment cluster *k*, with one of the five clusters serving as the reference category, *ULC*_*i*_ × *Cluster*_*ik*_, *UHC*_*i*_ × *Cluster*_*ik*_, *CC*_*i*_ × *Cluster*_*ik*_: interaction terms between each family care measure and employment-cluster dummies, *X*_*i*_: vector of control variables (gender, region (West or East Germany), education level, phased retirement, retirement timing, and number of months with missing information on family care), ε_*i*_: error term.

The estimates of the model represent conditional differences in pension entitlements associated with cumulative family care provision, relative to providing less or no care. With interaction terms included, these differences are interpreted within late-career employment clusters by comparing individuals who share similar employment trajectories but differ in their exposure to family care.

## Results

### Descriptive findings

Table 2 reports key characteristics of the analytical sample overall and separately for the late-career employment clusters. 58% of the sample are women. On average, sample members have accumulated 27 PEPs by the time they turn 55. Between ages 55 and 65, 34% provided any family care for at least one month. On average, around two months of credited care were provided, around nine months of uncredited low-intensity care and around two months of uncredited high-intensity care. Turning to the employment clusters, it becomes apparent that the clusters differ substantially in gender composition. Women are underrepresented in the cluster characterised by full-time employment (31%) but markedly overrepresented in the part-time employment (93%), unemployment/marginal employment (73%) and homemaker (98%) clusters. The early retirement cluster is most gender-balanced. Furthermore, before turning 55, individuals in the full-time employment cluster accumulate on average the highest number of PEPs (35 PEPs), followed by members of the early retirement cluster with 30 PEPs. In contrast, members of the homemaker cluster accumulate on average only 7 PEPs before turning 55. Although any family care for at least one month is present in all clusters, members of the early retirement and homemaker clusters show the highest rates, both exceeding 40%. Among credited caregivers, members of the homemaker cluster exhibit the longest average duration of caregiving, with almost 8 months. The average duration of uncredited low-intensity care varies from 7.6 months in the full-time employment cluster to approximately 11 months in the early retirement cluster. The average length of uncredited high-intensity care is shorter across all clusters but peaks in the early retirement cluster, amounting to 4.2 months. Table 4 in the Appendix provides details on further sample characteristics.

**Table 2:** Sample description

	All	Late-career employment clusters				
		Full-time employment	Part-time employment	Unemployment/Marginal employment	Early retirement	Homemaker
Share of women (in %)	58.5	30.6	92.5	73.3	52.7	98.4
Average PEPs accumulated before age 55	26.8	35.2	21.6	13.3	30.4	7.2
*Between ages of 55 and 65:*						
Share of persons providing ≥ 1 month of (any) family care (in %)	34.1	26.6	30.2	35.7	43.0	44.1
*Average duration (in months):*						
Uncredited low-intensity care	8.9	7.6	8.2	9.7	10.8	8.5
Uncredited high-intensity care	2.2	1.1	1.9	1.9	4.2	0.6
Credited care	1.9	0.3	1.3	3.0	2.3	7.9

Source: SOEP-RV 2020 v.2.0, own computations

### Regression results

**Fig. 1:**
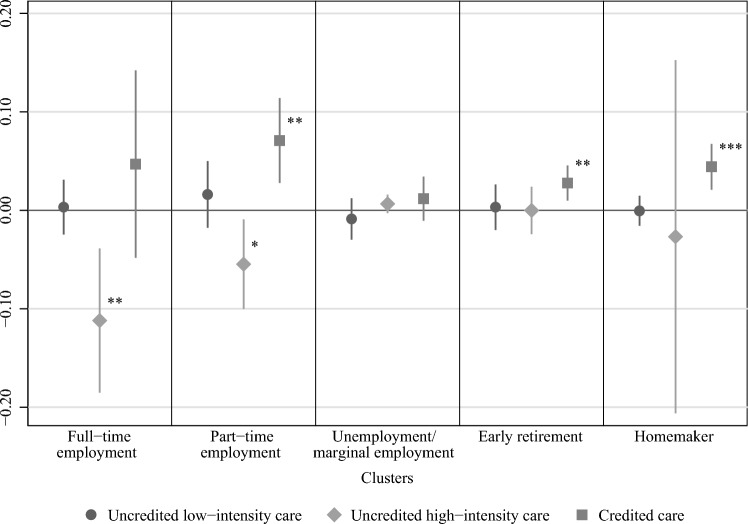
Adjusted mean changes in PEP accumulation between ages 55 and 65 for each additional month of family care within each employment cluster. Vertical error bars represent 95% confidence intervals. Within-cluster changes are based on a multivariate OLS regression including control variables for gender, region, education level, phased retirement, retirement timing, the number of months with missing information on family care. The OLS regression results are reported in Table 5 in the Appendix, and the within-cluster adjusted mean changes are presented in Table 6. *Source*: SOEP-RV 2020 v.2.0, own computations. Significance levels: * p < 0.05, ** p < 0.01, *** p < 0.001

Next, we aim to disentangle how late-career PEP accumulation differs between the different types of (non-)caregivers across different late-career employment clusters. Based on the OLS regressions, we estimated adjusted mean changes in PEP accumulation between ages 55 and 65 for each additional month of family care within each employment cluster, as illustrated in Fig. 1. Detailed OLS regression results and within-cluster adjusted mean changes based on them are presented in Tables 5 and 6 in the Appendix.

While uncredited low-intensity care shows no statistically significant association with PEP accumulation within any of the clusters, each additional month of uncredited high-intensity care is associated with lower PEP accumulation within the full-time (− 0.112, p < 0.01) and part-time employment clusters (− 0.055, p < 0.05). In contrast, each additional month of credited care is associated with higher PEP accumulation between 55 and 65 within the clusters characterised by early retirement (0.028, p < 0.01), homemaking (0.044, p < 0.001), and part-time employment (0.071, p < 0.01).

**Fig. 2:**
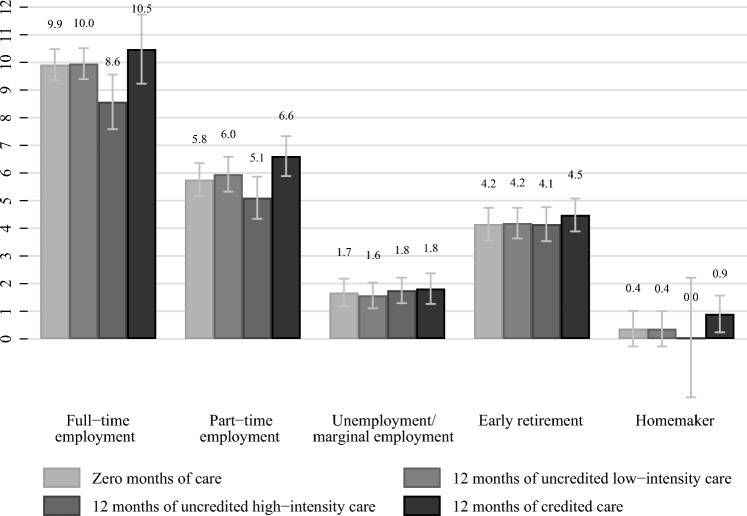
Within-employment cluster PEP accumulation predictions between ages 55 and 65, by care months. Vertical error bars represent 95% confidence intervals. Within-cluster predictions are based on a multivariate OLS regression including control variables for gender, region, education level, phased retirement, retirement timing, number of months with missing information on family care. Source: SOEP-RV 2020 v.2.0, own computations

To gain a deeper understanding of these findings, we visualised individuals’ predicted PEP accumulation between ages 55 and 65 within each employment cluster, comparing non-caregivers (0 months of care throughout ages 55–65) with caregivers providing 12 months of care, distinguishing uncredited low-intensity care, uncredited high-intensity care and credited care (Fig. 2). PEP accumulation differs remarkably between the clusters. While non-caregiving members of the full-time employment cluster accumulate, on average, around 9.9 PEPs, members of the homemaker cluster hardly accumulate any PEPs. Furthermore, as indicated by Fig. 1, for the full- and part-time employment clusters, uncredited high-intensity care duration is negatively associated with PEP accumulation: Members of the full-time employment cluster providing 12 months of uncredited high-intensity care accumulate, on average, 1.3 PEPs less than non-caregiving members (p < 0.01). For members of the part-time employment cluster, 12 months of uncredited high-intensity caregiving between ages 55 and 65 is associated with 0.7 less PEP accumulation compared to non-caregiving members (p < 0.05). For members of the early retirement, unemployment/marginal employment, and homemaker clusters, PEP accumulation is not linked to uncredited high-intensity care months.

Figure 2 also shows the positive association between credited care and PEP accumulation within the part-time employment, early retirement, and homemaker clusters depicted in Fig. 1. By providing credited care for 12 months, individuals in the part-time cluster accumulate, on average, 6.6 PEPs between 55 and 65 and 0.8 PEPs significantly more than those who did not provide care in this cluster (p < 0.01). Individuals in the early retirement cluster accumulate, on average, 0.3 PEPs more if they provide 12 months of credited care compared to cluster members who do not provide any care (p < 0.01), ending up with 4.5 PEPs accumulated between 55 and 65. Within the homemaker cluster, non-caregivers accumulate 0.4 PEPs between 55 and 65, while those providing credited care for 12 months accumulate 0.9 PEPs, a difference of 0.5 PEPs (p < 0.001). For members of the full-time employment and unemployment/marginal employment clusters, PEP accumulation is not linked to credited care months.

### Robustness checks

To examine the robustness of our results, we explored two alternative lengths for the SOEP family care variables (1) and (2) to address potential over-coding during the transformation from person-year to person-month data. For the first alternative measure, we reduced the length of the family care episode from twelve to six months, spanning from the second month before the interview month (t_0_) to the third month after. For the second alternative measure we took a stricter approach, shortening the episode to one month, considering only the interview month when family care was reported. Additional OLS regression analyses (Table S4.1 in the Supplementary Material) using the alternative measures yielded consistent directions and significance levels with the main results, indicating the robustness of the results across alternative transformations of family care information from person-year to person-month format.

## Discussion

Against the backdrop of population ageing and increasing (family) care needs, several European countries have introduced care credit schemes that aim to compensate for family care-related pension penalties (Van den Bosch et al. 2023; Eggers and Grages 2023). Yet eligibility is selective, leaving many caregivers uncredited. Moreover, prior research has rarely been able to assess pension accumulation through both credited and uncredited pathways. We address this gap by analysing late-career family caregiving, that is, between the ages of 55 and 65, and pension entitlement accumulation. Our study focuses on Germany, a context characterised by an employment-centred pension system and a family-based long-term care regime that includes a care credit scheme. Informed by a life-course perspective and prior research on the labour market consequences of providing family care, we asked (1) how late-career pension contribution accumulation differs between non-caregivers, credited caregivers, and uncredited caregivers across different late-career employment trajectories, and (2) to what extent pension care credits compensate for caregiving-related differences in pension contribution accumulation across late-career employment trajectories.

With regard to research question (1), we show that differences in pension accumulation vary both across caregiver types and across employment trajectories. Across late-career employment trajectories, uncredited low-intensity care is not associated with differences in accumulated pension earning points (PEPs). By contrast, uncredited high-intensity care is associated with lower PEP accumulation in two late-career employment trajectories characterised by mainly full-time and mainly part-time employment, respectively. Credited care, in turn, is associated with higher PEP accumulation among caregivers in the part-time employment, early retirement, and homemaker clusters. Notably, the part-time and homemaker clusters are predominantly composed of women.

The results on uncredited low-intensity care suggest the absence of long-term pension disadvantages and are in line with previous research showing that low-intensity care has little or no impact on the intensive or extensive margin of paid work (e.g. Lilly et al. 2007; Moussa 2019), although some evidence indicates transitions from full-time to part-time employment among low-intensity caregivers (Ehrlich 2023; Kelle 2020). This may indicate that individuals providing uncredited low-intensity care, that is, one to two hours of care per day, make only limited adjustments to paid work and may instead reallocate time from other domains, such as leisure.

The lower PEP accumulation associated with uncredited high-intensity care in the part-time and full-time employment clusters is consistent with evidence that high-intensity caregiving is more likely to trigger reductions in working hours or labour market exits (e.g. Lilly et al. 2007; Moussa 2019). In addition, employment disruptions may have longer-run consequences, such as foregone promotions, transitions to less-demanding jobs, or being trapped in part-time or non-employment even after family caregiving ends (Ehrlich et al. 2020; Keck 2016), which can further depress pension accumulation. By contrast, among clusters characterised by weaker labour market attachment, we observe neither losses nor gains in PEP accumulation associated with uncredited high-intensity care, which is consistent with a lower potential for work-care conflict in these trajectory clusters.

The PEP gains among credited caregivers in the part-time employment, early retirement, and homemaker clusters are in line with previous research based on administrative pension records, which documents compensatory, and in some cases positive, pension implications of credited caregiving, particularly among groups with weaker or no labour market attachment (e.g. Czaplicki 2016). These gains are plausibly linked to the design of the care credit scheme, which, among other eligibility criteria, targets caregivers with limited paid work, that is, up to 30 h per week, but also retirees.

This brings us to research question (2), namely to what extent pension care credits compensate for caregiving-related differences in pension contribution accumulation across late-career employment trajectories. Overall, our findings point to selective and uneven compensation. In trajectories characterised by labour market detachment, such as early retirement and homemaking, credited caregivers accumulate more PEPs than non-caregivers. At the same time, uncredited high-intensity caregiving is associated with lower PEP accumulation in trajectories with high labour-market attachment, particularly in the full-time cluster. Importantly, the part-time cluster illustrates the scheme’s selectivity most clearly: while credited caregivers in this trajectory show PEP gains, uncredited high-intensity caregivers following a part-time trajectory experience PEP losses relative to non-caregivers. One plausible explanation is that credited caregivers in this trajectory are selectively those who meet the scheme’s eligibility criteria (e.g. working no more than 30 h per week or fulfilling care-recipient requirements) and have actively applied for them. In these cases, care credits operate as a pension ‘add-on’ alongside part-time employment. By contrast, high-intensity caregivers who remain uncredited may experience earnings-related contribution losses without any compensatory support. Taken together, care credits do not systematically reach those caregivers and trajectory contexts in which pension losses appear most pronounced, which is consistent with the scheme’s eligibility rules, including the requirement that caregiving is combined with limited paid work and care-recipient eligibility conditions. Although we cannot identify the precise reasons why some high-intensity caregivers remain uncredited, the pattern raises equity concerns because caregivers with comparable care intensity may be treated differently depending on eligibility and involvement in paid work.

Even when caregivers receive care credits, the magnitude of credited PEPs is modest, suggesting that care credits function more as partial mitigation than as full compensation. For example, Fig. 2 indicates that homemakers who provide credited care for 12 months accumulate, on average, 0.5 more PEPs than homemakers who do not provide care. At the current PEP value (€40.79), this corresponds to about €20.40 more per month in retirement. By comparison, one year of employment at average earnings yields 1 PEP. However, homemakers’ average cumulative PEPs by age 55 are low (7.2 PEPs; see Table 1), so these additions are unlikely to substantially alter pension entitlements.

Some limitations should be noted. We know that family care in working age is mainly provided by women (Kelle and Ehrlich 2024). Consequently, our results are likely to be mainly driven by the women in the sample. This is particularly relevant for our findings on late-career paths with little or no labour market attachment, which are disproportionately dominated by women. However, gender-stratified analyses were not feasible due to sample size constraints, leaving gendered consequences of care dynamics and late-career trajectories an important area for future research. Moreover, it would be important to examine interactions between gender and region, as women’s employment careers differ across the cohorts studied, with East German women generally exhibiting stronger labour market attachment than West German women. Due to limited case numbers, however, such analyses were not possible in the present study.

Furthermore, it could be argued that our findings on PEP accumulation in the full-time and part-time employment clusters in response to family care may partly reflect selective processes associated with differences in family care take-up across socio-economic status (SES), rather than career adjustments in response to care alone. While we aimed to minimise bias from potential endogeneity between family care and paid work by using a within-cluster analysis strategy and controlling for education level, SES may still shape work–care reconciliation among caregivers. Evidence on an SES gradient in family care take-up remains inconclusive (e.g. Albertini et al. 2023; Alonso-Perez et al. 2024; Kelle and Ehrlich 2024; Quashie et al. 2022), and prior research shows that career adjustments occur in response to family care even when controlling for SES (e.g. Ehrlich 2023; Kelle 2020). Future analyses could explore SES heterogeneity within clusters to more fully disentangle its role in the relationship between family care and PEP accumulation.

Despite these limitations, our study makes three contributions. First, by measuring pension entitlements, we provide direct quantitative evidence on the longer-term pension implications of late-career family caregiving, going beyond what can be inferred from studies focussing primarily on short-term labour market outcomes. Second, we extend the limited long-term literature by distinguishing credited and uncredited caregiving and differentiating care intensity among the uncredited. Leveraging the SOEP-RV linkage, we make visible heterogeneity in caregiving-related pension accumulation and gaps in credit coverage that prior studies typically could not assess due to data limitations. Third, by embedding caregiving within late-career employment trajectories, we show that caregiving-related pension differences are conditioned by the employment context. Substantively, our findings suggest that care credits have selective coverage and are modest in magnitude, and that they do not systematically reach those caregivers and employment contexts in which pension losses appear most pronounced, pointing to distributional and equity implications across trajectories.

## Conclusion

Overall, our findings show that late-career family caregiving can have longer-term pension implications that complement the more widely studied short-term labour market consequences, such as reductions in working hours or earnings. At the same time, these pension consequences are heterogeneous and shaped by policy design. In Germany, pension care credits provide only modest protection and are granted selectively, so that high-intensity caregiving can still translate into pension penalties in employment contexts where contributions are most at stake. This is particularly salient given that pensions are the main source of income in later life for most individuals, and that women, who face higher risks of old-age poverty, remain more likely to provide family care, while policy increasingly emphasises longer working lives and higher labour force participation, particularly among women. Taken together, these findings raise the question of whether long-term care policy should place greater emphasis on supporting caregivers who provide home-based family care while remaining in paid work, given policy priorities of both family-provided care and high labour force participation.

## Electronic supplementary material

Below is the link to the electronic supplementary material.

Supplementary file1 (DOCX 267 KB)

## Data Availability

The administrative records of the German Pension Insurance (RV) and the German Socio-Economic Panel data (SOEP) must be ordered separately and are then linked by the user. The SOEP-RV data link used in the analyses (SOEP-RV VSKT 2020, v. 2.0, 10.5684/soep.v37-RV.VSKT2020.V2.0) can be ordered at the RV Research Data Center (https://fdz-rv.de/en/data-use/overview). The SOEP data used in the analyses (SOEP v37, EU Edition, 10.5684/soep.core.v37eu) can be ordered at the SOEP Research Data Center (https://www.diw.de/en/diw_01.c.601584.en/data_access.html).

## Appendix

See Tables 3, 4, 5 and 6.

**Table 3:** Description of variables’ operationalisation and descriptive statistics

Variables	Source	Operationalisation	M/Prop	SD
*Dependent variable:*				
Accumulated PEP	*RV*	Continuous variable: individual monthly accumulated PEPs between ages 55 (660th age month) and 65 (780th age month)	5.98	5.65
*Independent variables:*				
Uncredited low-intensity care^1^	*SOEP*	Continuous variable: number of months between ages 55 and 65 in which family care was provided for up to 10 h/workweek	8.72	17.81
Uncredited high-intensity care^1^	*SOEP*	Continuous variable: number of months between ages 55 and 65 in which family care was provided for more than 10 h/workweek	2.12	9.02
Credited care	*RV*	Continuous variable: number of months between ages 55 and 65 in which PEPs were received for care	1.93	10.21
Late-career employment clusters^2^	*SOEP* *RV*	Categorical variable typifying an individual’s employment career between 55 and 65:		
		(1) full-time employment	0.35	
		(2) early retirement	0.27	
		(3) homemaker	0.07	
		(4) unemployment/marginal employment	0.11	
		(5) part-time employment	0.20	
*Control variables:*				
Gender	*SOEP*	Dummy variable: (0) male, (1) female	0.58	
Education level	*SOEP*	Categorical variable representing the highest educational attainment up to age 65:		
		(1) low level of education (ISCED 0–2),	0.08	
		(2) intermediate level of education (ISCED 3–4),	0.63	
		(3) high level of education (ISCED 5–6)	0.29	
Region	*SOEP*	Dummy variable indicating whether the individual has predominantly lived in East or West Germany between ages 55 and 65: (0) East Germany, (1) West Germany	0.30	
Retirement timing	*SOEP* *RV*	Categorical variable indicating individual’s age(-month) at retirement:		
		(1) age < 60,	0.07	
		(2) age ≥ 60 & ≤ 65,	0.62	
		(3) age > 65	0.31	
Phased retirement	*RV*	Dummy variable indicating whether individual phased gradually into retirement: (0) no phased retirement, (1) phased retirement	0.14	
Missing care information^3^	*SOEP* *RV*	Continuous variable: number of months between ages 55 and 65 without family care information	16.98	29.66

^1^Since the dependent variable and most other relevant variables are measured in or based on person-months, while low-intensity and high-intensity care are measured on a person-year basis, they must be converted into a person-month format. This is done as follows: if respondents reported uncredited low-intensity care in one panel wave t_0_, uncredited low-intensity care lasts from the fifth month before the month of interview in t_0_ until the sixth month after the month of interview. If individuals reported uncredited low-intensity care in two subsequent panel waves t_0_ and t_1_, uncredited low-intensity care is stretched from the fifth month before the month of interview in t_0_ to the sixth month after the month of interview in t_1_ and so on

^2^This categorisation results from a cluster sequence analysis based on individuals’ monthly employment spell data between ages 55 and 65. Further details on how the variable was constructed can be found in Section S1 in the Supplementary Material

^3^The SOEP family care measures had missing information in 16,867 out of 98,494 person-months, i.e. 17.1 per cent, from n = 556 persons between the ages of 55 and 65. In 75 per cent of cases, the gaps were no longer than 16 person-months. Gaps at the beginning, i.e. left-censored spells (n = 6,968 person-months), or at the end, i.e. right-censored spells (n = 4,798 person-months), in the family care biography remained. Within-gaps (n = 3,044 person-months) were filled with available information before and after the gap. If the family care status before the gap is the same as the family care status after the gap, the gap is filled with the corresponding family care status. If the family care status before the gap is different from the family care status after the gap, the gap is divided into two equal parts. The first part is assigned the family care status before the gap, the second part the family care status after the gap. If the gaps last for an uneven number of months, the remainder month is assigned to the first part. After the procedure, 13,823 person-months of 363 individuals remained without information, i.e. 14.03 per cent of all person-month family care information. Missing family care information is controlled for in the analyses

**Table 4:** Descriptive statistics

	Late-career employment clusters									
	Full-time employment		Part-time employment		Unemployment/ Marginal employment		Early retirement		Homemaker	
	*M/Prop*	*SD*	*M/Prop*	*SD*	*M/Prop*	*SD*	*M/Prop*	*SD*	*M/Prop*	*SD*
Accumulated PEP	10.67	5.29	5.09	3.40	0.73	0.76	4.18	4.65	0.48	1.22
Uncredited low-intensity care	7.36	17.06	8.09	18.43	9.48	20.39	10.78	17.74	8.20	15.65
Uncredited high-intensity care	1.04	4.73	1.90	7.76	1.88	11.50	4.17	12.95	0.59	2.61
Credited care	0.34	2.62	1.34	6.19	3.02	12.58	2.34	11.42	7.87	22.85
Gender (Woman)	0.31		0.93		0.73		0.53		0.98	
Education level (low)	0.03		0.08		0.20		0.09		0.15	
Education level (intermediate)	0.63		0.60		0.70		0.62		0.69	
Education level (high)	0.34		0.32		0.10		0.30		0.16	
Region (East Germany)	0.28		0.32		0.34		0.37		0.02	
Retirement timing (age < 60)	0.00		0.04		0.00		0.23		0.00	
Retirement timing (age ≥ 60 & ≤ 65)	0.61		0.76		0.43		0.75		0.10	
Retirement timing (age > 65)	0.39		0.20		0.57		0.02		0.90	
Phased retirement (Yes)	0.14		0.10		0.00		0.26		0.00	
No. of months with missing care information	19.57	32.69	15.40	28.11	21.98	33.09	12.74	23.37	17.48	32.63
N	284		161		86		222		61	

*Source* SOEP-RV 2020 v.2.0, own computations. M = mean; Prop = proportion; SD = standard deviation

**Table 5:** Full OLS regression on PEP accumulation (ages 55–65)

	b (se)
Uncredited low-intensity care (*in months*)	0.003
	(0.014)
Uncredited high-intensity care (*in months*)	− 0.112^**^
	(0.037)
Credited care (*in months*)	0.047
	(0.049)
Clusters (*Ref. Full-time employment cluster*)	
Part-time employment cluster	− 4.157^***^
	(0.434)
Unemployment/Marginal employment cluster	− 8.240^***^
	(0.404)
Early retirement cluster	5.766^***^
	(0.411)
Homemaker cluster	− 9.545^***^
	(0.479)
Cluster × Uncredited low-intensity care (*Ref. Full-time employment × Uncredited low-intensity care*)	
Part-time employment cluster × Uncredited low-intensity care	0.013
	(0.022)
Unemployment/Marginal employment cluster × Uncredited low-intensity care	− 0.012
	(0.018)
Early retirement cluster × Uncredited low-intensity care	− 0.000
	(0.019)
Homemaker cluster × Uncredited low-intensity care	− 0.004
	(0.016)
Cluster × Uncredited high-intensity care (*Ref. Full-time employment cluster × Uncredited high-intensity care*)	
Part-time employment cluster × Uncredited high-intensity care	0.057
	(0.044)
Unemployment/Marginal employment cluster × Uncredited high-intensity care	0.119^**^
	(0.037)
Early retirement cluster × Uncredited high-intensity care	0.112^**^
	(0.039)
Homemaker cluster × Uncredited high-intensity care	0.085
	(0.098)
Cluster × Credited care (*Ref. Full-time employment × Credited care*)	
Part-time employment cluster × Credited care	0.024
	(0.054)
Unemployment/Marginal employment cluster × Credited care	− 0.035
	(0.050)
Early retirement cluster × Credited care	− 0.019
	(0.050)
Homemaker cluster × Credited care	− 0.003
	(0.050)
Gender (*Ref. Male*)	
Female	− 1.532^***^
	(0.298)
Region (*Ref. East Germany*)	
West Germany	0.788^**^
	(0.279)
Education level (*Ref. Low*)	
Intermediate level	0.615^+^
	(0.330)
High level	3.136^***^
	(0.442)
Phased retirement *(Ref. no phased retirement)*	
Phased retirement	4.415^***^
	(0.395)
Retirement timing (*Ref. age* ≥ *60 &* ≤ 65)	
age < 60	− 1.165^**^
	(0.430)
age > 65	1.797^***^
	(0.389)
Missing family care information (*in months*)	− 0.010^*^
	(0.004)
Constant	8.038^***^
	(0.498)
*R* ^2^	0.625
N	814

*Source* SOEP-RV 2020 v.2.0, own computations. Significance levels: + *p* < 0.1, * *p* < 0.05, ** *p* < 0.01, *** *p* < 0.001. Standard errors in parentheses

**Table 6:** Postestimation results from OLS regression (Table 5): Adjusted mean changes in PEP accumulation between ages 55 and 65 by family care intensity within each employment cluster

	Model 1
*Clusters × Uncredited low-intensity care*	
Full-time employment cluster × Uncredited low-intensity care	0.003
	(0.014)
Part-time employment cluster × Uncredited low-intensity care	0.016
	(0.017)
Unemployment/marginal employment cluster × Uncredited low-intensity care	− 0.009
	(0.011)
Early retirement cluster × Uncredited low-intensity care	0.003
	(0.012)
Homemaker cluster × Uncredited low-intensity care	− 0.000
	(0.008)
*Clusters × Uncredited high-intensity care*	
Full-time employment cluster × Uncredited high-intensity care	− 0.112^**^
	(0.037)
Part-time employment cluster × Uncredited high-intensity care	− 0.055^*^
	(0.023)
Unemployment/marginal employment cluster × Uncredited high-intensity care	0.007
	(0.005)
Early retirement cluster × Uncredited high-intensity care	− 0.000
	(0.012)
Homemaker cluster × Uncredited high-intensity care	− 0.027
	(0.091)
*Cluster × Credited care*	
Full-time employment cluster × Credited care	0.047
	(0.049)
Part-time employment cluster × Credited care	0.071^**^
	(0.022)
Unemployment/marginal employment cluster × Credited care	0.012
	(0.011)
Early retirement cluster × Credited care	0.028^**^
	(0.009)
Homemaker cluster × Credited care	0.044^***^
	(0.012)
*R* ^2^	0.625
N	814

*Source* SOEP-RV 2020 v.2.0, own computations. Significance levels: + *p* < 0.1, * *p* < 0.05, ** *p* < 0.01, *** *p* < 0.001. Standard errors in parentheses. Controlled for gender, education, region, retirement timing, phased retirement, number of months with missing family care information. These results are displayed in Fig. 1

## Footnotes

1 While the German pension system also comprises occupational and private components, this study focuses on the statutory public pension scheme. On average, these additional components are less important as sources of income in later life, and unlike the statutory scheme, they do not recognise family caregiving through pension care credits.

2 Up to 2016, persons received pension credits if they provided care to a long-term care insurance beneficiary in the home environment for a minimum of 14 h/week and did not work for more than 30 h/week.

3 The reported range of 0.18–0.94 PEPs is an approximate author calculation based on Deutsche Rentenversicherung (2023, 2024). We convert the additional monthly pension amount credited for one year of caregiving in 2023 (*Rentenbetrag pro Monat*; Deutsche Rentenversicherung 2023) into PEPs by dividing it by the 2023 pension value (*Rentenwert*; Deutsche Rentenversicherung 2024). Full calculation details are available from the authors upon request.

4 We allowed for up to 36 months of missing information in the employment biography. Details on how we dealt with missing information in the employment and family care biographies can be found in Section S3 of the Supplementary Material and in the Appendix.

5 Phased retirement (*Altersteilzeit*) is a work arrangement where employees aged 55 or older, in agreement with their employer, can gradually reduce their work time before transitioning into retirement. Most commonly, the time before retirement is divided into two equal phases: one of normal work with half salary, and another phase where the employee is officially retired but still receives half their salary from the first phase. In the context of our analysis, it is important to account for the group of individuals in this work arrangement before retirement because, although they are retired during the aforementioned phase, these individuals continue to accumulate PEPs.
