# Supplementary material for: Is there a pension penalty for late-career family caregiving in Germany? The role of pension care credits and employment career
Source: Eur J Ageing. 2026 Jul 14;23(1):32. doi: 10.1007/s10433-026-00923-y (PMC13365077; doi:10.1007/s10433-026-00923-y)
Supplement: Supplementary file 1 — Supplementary file1 (DOCX 267 KB) [file 10433_2026_923_MOESM1_ESM.docx]

Supplementary Material

**Section S1: Sample selection steps**

*Figure S1.1 Sample selection flowchart*


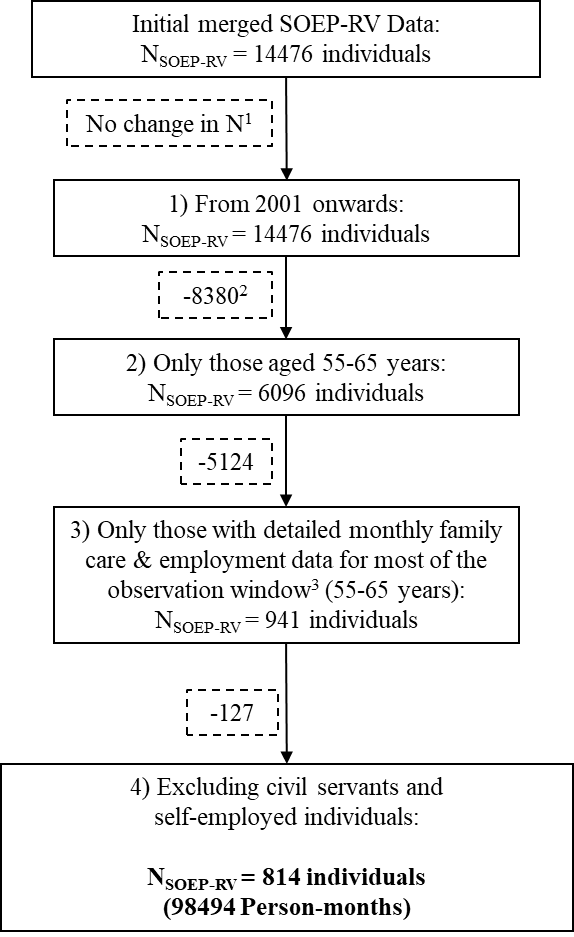


^1^In the first step, excluding information prior to 2001 does not reduce the number of individuals in our sample. This is because consent for linking SOEP and pension insurance (RV) records was obtained in 2020. Consequently, all individuals in our initial dataset must have participated in the SOEP after 2001. Nevertheless, this step does exclude observations (i.e., person-months, as the dataset is structured in monthly format) before 2001.

^2^In the second step, restricting the sample to individuals aged between 55 and 65 leads to the exclusion of 8,380 individuals. The majority (96.4%) are younger than 55 years, while 3.6% are individuals who were already older than 65 years at some point between 2001 and 2020.

^3^In the third step, having “data for most of the observation window” is defined as having employment and family care information for at least 7 years out of the 10 years observed between ages 55 and 65.

^4^Individuals who were identified as civil servants or self-employed for more than 60 per cent of their careers, or who received a civil servant pension, were excluded. These groups are not covered by statutory pension insurance, or only to a low degree, and their inclusion could therefore bias our analyses.

**Section S2: Assessment of Sample Selectivity**

*Table S2.1 Comparison of characteristics at age 55 across SOEP samples*

| **Characteristics at Age 55** | **Analytical sample** | **SOEP-RV sample** | **SOEP sample** |
| --- | --- | --- | --- |
| **Accumulated pension earning points (mean)** | 26.84 | 24.95 | - |
| **Gender** |  |  |  |
| Men | 41.5% | 45.3% | 45.3% |
| Women | 58.5% | 54.7% | 54.7% |
| **Region** |  |  |  |
| East Germany | 30.2% | 31.2% | 25.4% |
| West Germany | 69.8% | 68.8% | 74.6% |
| **Education (ISCED)** |  |  |  |
| Low education | 8.4% | 10.8% | 10.7% |
| Middle education | 63.4% | 61.5% | 61.1% |
| High education | 28.3% | 27.7% | 28.2% |
| **Number of individuals aged 55 years (birth cohorts 1946–1955)** | 814 | 1,882 | 2,890 |

Notes: The analytical sample consists of individuals from birth cohorts 1946–1955 resulting from the selection steps described in Section S1. The SOEP-RV sample includes all individuals aged 55 in those cohorts with linked SOEP-RV data. The SOEP sample includes all individuals aged 55 in the relevant cohorts, regardless of data linkage.

Source: SOEP-RV 2020 v.2.0 and SOEP v37, own computations.

**Section S3: Generating the late-career employment trajectory clusters**

For generating the cluster variable presented in the independent variables section of the paper, we use sequence and cluster analysis (e.g., Raab & Struffolino, 2022). The variable is based on monthly employment spell data provided by the SOEP activity calendar as well as by administrative records from RV data. SOEP respondents annually report their employment status for each month of the previous year in the activity calendar. A matrix is presented displaying a list of possible employment statuses (rows) and all months in the previous calendar year (columns). Respondents are then asked to check which of the employment statuses listed in Table S3.1 (middle column) applied to them in each month. Fourteen different employment statuses could be chosen for the observation period (2001–2020), while for this study’s population three employment statuses were excluded as not empirically relevant (school/university/vocational school; first-time in-service training or apprenticeship; parental leave). However, we also supplemented SOEP information with person-month RV data (Table S3.1, right-hand column).

*Table S3.1: Recoding of SOEP and RV employment spells*

| Recoding of SOEP-RV employment states | Original SOEP activity calendar employment status | Original RV employment status |
| --- | --- | --- |
| (1) Old-age retirement | Retirement | Pension receipt |
| (2) Early retirement^1^ | - | Receipt of occupational disability or reduced earnings capacity pension and waiting period prior to receipt |
| (3) Full-time employment | Full-time employment |  |
| (4) Part-time employment | Part-time employment |  |
| (5) Marginal employment^2^ | Mini-Job (since 2004) | Marginal employment with and without compulsory insurance contributions (2001–2003) |
| (6) Unemployment | Registered as unemployed |  |
| (7) Homemaking | Homemaker |  |
| (8) Other | Vocational training (in-company)  Short-time working hours^3^  Military service/  community service/  social-ecological year/  federal volunteer service    Further training/retraining /  further occupational training    Other |  |

^1^This status refers to those months before official retirement when people are already considered unable to work for pay due to occupational disability or reduced earnings capacity.

^2^This status refers to months in which individuals worked in marginal employment (German: *geringfügige Beschäftigung*), labelled “Mini-Job” since 2003. Marginal employment or a Mini-Job is generally described as part-time and low-wage employment (up to 2002: € 325/month; 2003–2012: € 400/month; since 2013: € 450/month). This employment relationship is exempted from income tax and subject to reduced social security contributions. Moreover, in the case of marginal employment, individuals can apply for exemption from contributions to the German pension insurance. As a result, the number of PEPs accrued is generally low or non-existent.

^3^Short-time work is mostly initiated by the employer due to economic crisis situations.

Since respondents can report multiple employment statuses in the same month in the monthly activity calendar, activity statuses may overlap. For example, a respondent may report being retired but also being a homemaker in the same month. A requirement of sequence cluster analysis is that (employment) statuses are mutually exclusive. Therefore, overlapping information must be resolved. To achieve this, a hierarchy of activity statuses was established that prioritizes certain activity statuses over others (as shown by the order of the list items in Table S3.1). ‘Retirement’ is given the highest priority and ‘other’ status the lowest. Because retirement is given the highest priority, a respondent who, for example, is both a retiree and engaged in other activities in the same month is classified only as retired.

Moreover, our employment data are not without missing information. Between the ages of 55 (660^th^ age month) and 65 (780^th^ age month) 12.5 percent of all person-month observations had missing information (12,366 person-months from 378 individuals). In order to fill the gaps, we used information from person-year employment or labour force status information from the SOEP *pgen-*dataset, or person-month employment status information from RV data. This procedure filled most of the missings. The rest of the missings (267 person-months, which is less than 0.3 percent of all person-month observations) were filled with either neighbouring information or with person-year information from the SOEP *pbiospe*-dataset.

Once the employment biographies between 55 and 65 years (121 age-months) were ready to be analysed, we conducted the sequence and cluster analysis. Firstly, in line with other scholars (Carmichael & Ercolani, 2016; Czaplicki, 2020; Rowold et al., 2022) we calculated substitution costs for the job sequences using the TRATE method, which relied on transition probabilities between jobs to estimate the substitution costs. Next, we computed pairwise distances between job sequences using the Optimal Matching (OM) algorithm, considering an indel cost of 1 and the calculated substitution costs from the observed transition rates. By utilizing these methods, we gained valuable insights into the similarity or dissimilarity of job transitions, facilitating the identification of distinct trajectory patterns within the data. Subsequently, we conducted a hierarchical clustering analysis using the Ward linkage method on the obtained pairwise distances and, based on cluster quality indicators, we chose the five-cluster solution that maximised the Average Silhouette Width (0.5) and achieved an R^2^ of 0.49 (see Figure S3.1). Cluster sequence analysis was done with the R packages TraMineR and TraMineRextras (Gabadinho et al., 2011; Ritschard et al., 2024).

Figure S3.2 plots the sequence of state distributions, that is, the frequencies of the states in each month, between ages 55 and 65 for each cluster resulting from the five-cluster solution. After considering the information presented in Table 1 in the manuscript and Figure S3.2, we named the five clusters on the basis of the most frequent employment status within the analysed employment biographies. Looking at Figure S3.2, we see, for instance, that the full-time employment cluster is dominated by full-time employment up to the age of 63 years, when the share of retirement status slowly increases.

*Figure S3.1: Goodness-of-fit statistics for 2–15 clusters*


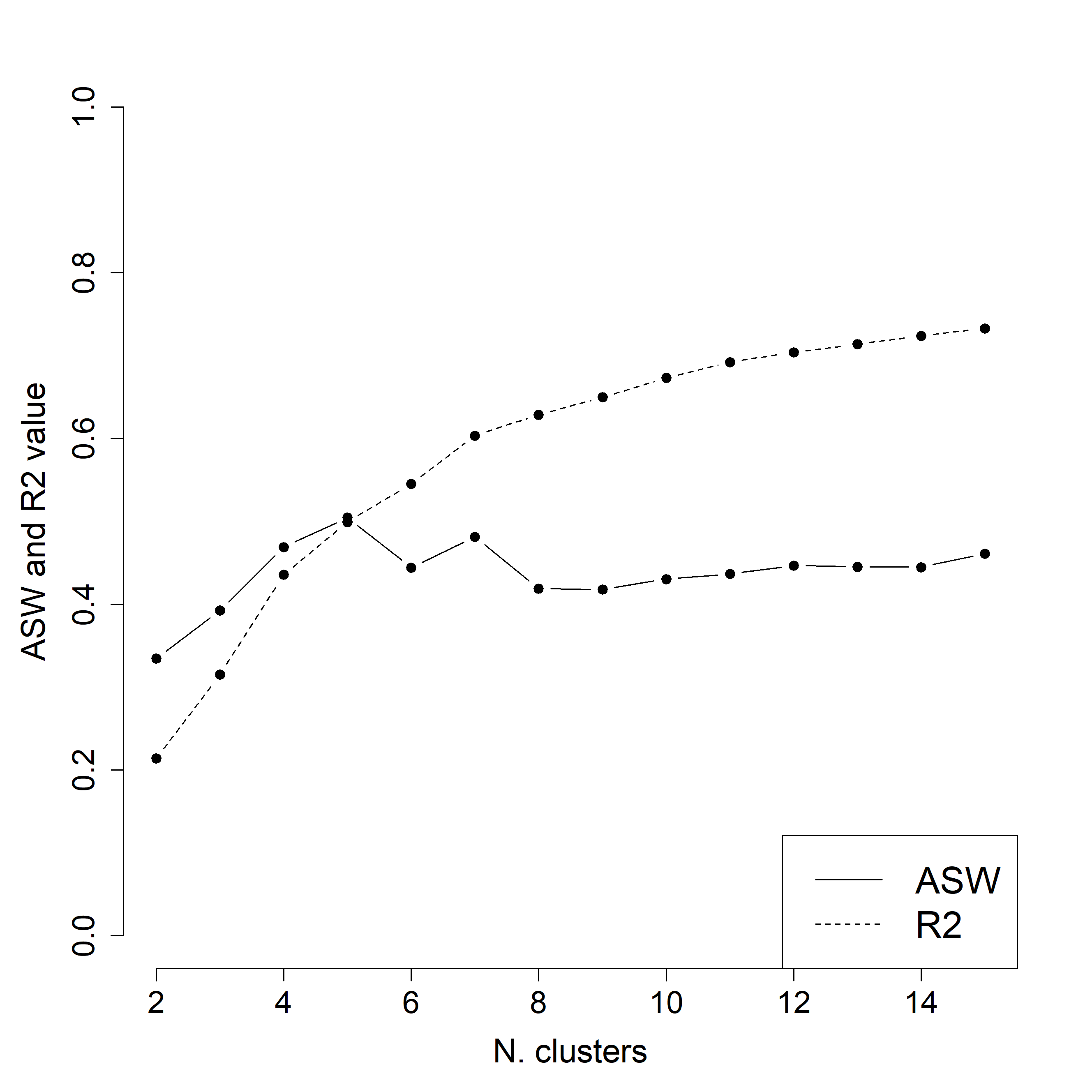


Source: SOEP-RV 2020 v.2.0, own computations

*Figure S3.2: State distribution plot of five employment clusters*


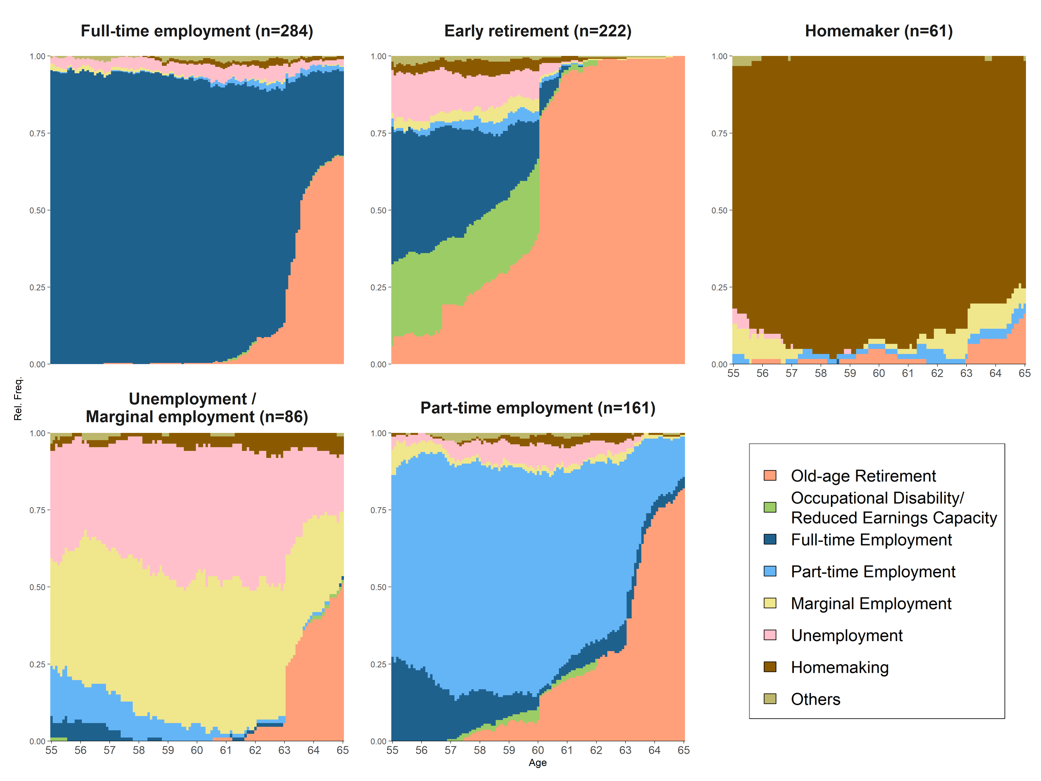
 Source: SOEP-RV 2020 v.2.0, own computations

**Section S4: Sensitivity analyses**

*Sensitivity Analysis: Alternative lengths of family care duration.* In Table S4.1, we contrast the postestimation results for the interaction terms from three OLS regression analysis models, each including SOEP family care variables with alternative lengths. Model 1 shows the results of the main analyses presented in the article with the annual SOEP family care information stretched to a twelve-month period. In Model 2, the annual SOEP family care information is stretched to a six-month period; in Model 3, the annual SOEP family care information is transformed to a one-month period.

*Table S4.1:* *Postestimation results from* *OLS regressions on PEP accumulation between the ages of 55 and 65 using alternative lengths of family care duration (annual information stretched to 12 months (Model 1), 6 months (Model 2) and 1 month (Model 3)*

|  | Model 1 | Model 2 | Model 3 |
| --- | --- | --- | --- |
|  |  |  |  |
| *Clusters x Uncredited low-intensity care* |  |  |  |
| Full-time employment cluster x Uncredited low-intensity care | 0.003 | 0.007 | 0.064 |
|  | (0.014) | (0.015) | (0.176) |
| Part-time employment cluster x Uncredited low-intensity care | 0.016 | 0.016 | 0.187 |
|  | (0.017) | (0.018) | (0.199) |
| Unemployment/marginal employment cluster x Uncredited low-intensity care | -0.009 | -0.009 | -0.096 |
|  | (0.011) | (0.012) | (0.131) |
| Early retirement cluster x Uncredited low-intensity care | 0.003 | 0.004 | 0.029 |
|  | (0.012) | (0.013) | (0.141) |
| Homemaker cluster x Uncredited low-intensity care | -0.000 | -0.002 | -0.061 |
|  | (0.008) | (0.008) | (0.105) |
|  | |  |  |
| *Clusters x Uncredited high-intensity care* | |  |  |
| Full-time employment cluster x Uncredited high-intensity care | -0.112^**^ | -0.154^**^ | -1.459^*^ |
|  | (0.037) | (0.048) | (0.572) |
| Part-time employment cluster x Uncredited high-intensity care | -0.055^*^ | -0.067^*^ | -0.700^*^ |
|  | (0.023) | (0.028) | (0.317) |
| Unemployment/marginal employment cluster x Uncredited high-intensity care | 0.007 | 0.006 | 0.096 |
|  | (0.005) | (0.004) | (0.062) |
| Early retirement cluster x Uncredited high-intensity care | -0.000 | -0.001 | -0.015 |
|  | (0.012) | (0.013) | (0.156) |
| Homemaker cluster x Uncredited high-intensity care | -0.027 | -0.049 | -0.188 |
|  | (0.091) | (0.105) | (1.394) |
|  |  |  |  |
| *Clusters x Credited care* |  |  |  |
| Full-time employment cluster x Credited care | 0.047 | 0.051 | 0.028 |
|  | (0.049) | (0.043) | (0.059) |
| Part-time employment cluster x Credited care | 0.071^**^ | 0.074^***^ | 0.061^**^ |
|  | (0.022) | (0.022) | (0.021) |
| Unemployment/marginal employment cluster x Credited care | 0.012 | 0.012 | 0.017 |
|  | (0.011) | (0.011) | (0.011) |
| Early retirement cluster x Credited care | 0.028^**^ | 0.028^**^ | 0.027^**^ |
|  | (0.009) | (0.009) | (0.010) |
| Homemaker cluster x Credited care | 0.044^***^ | 0.044^***^ | 0.045^***^ |
|  | (0.012) | (0.012) | (0.011) |
| *R*^2^ | 0.625 | 0.623 | 0.624 |
| Observations | 814 | 814 | 814 |

Notes: Standard errors in parentheses. Controlled for gender, education, region, retirement timing, phased retirement, and total number of months without care information. Model 1 uses annual SOEP family care data stretched to 12 months. Model 2 adjusts it to 6 months, and Model 3 to 1 month.

Source: SOEP-RV 2020 v.2.0, own computations. Significance levels: + p < 0.1, * p < 0.05, ** p < 0.01, *** p < 0.001.

**References**

Carmichael, F., & Ercolani, M. G. (2016). Unpaid caregiving and paid work over life-courses. Different pathways, diverging outcomes. *Social Science & Medicine*, *156*, 1-11. https://doi.org/10.1016/j.socscimed.2016.03.020

Czaplicki, C. (2020). *Die Kombination von Pflege- und Erwerbstätigkeit im Lebensverlauf. Strukturen, Muster und Pfadabhängigkeiten*. Springer VS. https://doi.org/10.1007/978-3-658-30089-0

Gabadinho, A., Ritschard, G., Müller, N. S., & Studer, M. (2011). Analyzing and Visualizing State Sequences in R with TraMineR. *Journal of Statistical Software*, *40*(4), 1 - 37. https://doi.org/10.18637/jss.v040.i04

Raab, M., & Struffolino, E. (2022). *Sequence Analysis*. SAGE.

Ritschard, G., Studer, M., Bürgin, R., Liao, T., Gabadinho, A., Fonta, P., Müller, N., & Rousset, P. (2024). *Package TraMineRextras*.

Rowold, C., Struffolino, E., & Fasang, A. E. (2022). Life-course-sensitive analysis of group inequalities: Combining Sequence Analysis with the Kitagawa-Oaxaca-Blinder decomposition. https://doi.org/10.31235/osf.io/7k4vt
